# Ethnicity of Referrals to Liaison Psychiatry Services at Aberdeen Royal Infirmary: An Audit

**DOI:** 10.1192/bjo.2025.10538

**Published:** 2025-06-20

**Authors:** Hira Ahmad, Murray Smith

**Affiliations:** 1 University of Aberdeen, Aberdeen, United Kingdom; 2 Liaison Psychiatry Department, Aberdeen Royal Infirmary, Aberdeen, United Kingdom

## Abstract

**Aims:** This audit aims to record the ethnicity of referrals to Liaison Psychiatry from January 1 to December 31, 2024, to evaluate whether the ethnic representation of these referrals aligns with the demographic composition of the local population and to identify any disproportionality in certain ethnic groups, which may require targeted intervention or further investigation.

**Methods:** Electronic records of all Liaison Psychiatry referrals made between January 1 and December 31, 2024, were reviewed. 539 patients were referred for self-harm and 607 patients were ward referrals. Emergency and ward referrals were grouped under ‘ward referrals’. Data from electronic records were cross-referenced with paper records to ensure accuracy. Ethnicity data, where missing, were retrieved from SCI-Docs when possible. Referral ethnicity data were compared with 2024 census data from four constituencies. All analyses adhered to strict confidentiality protocols, ensuring anonymity and privacy for all patients.

**Results:** Between January 1 and December 31, 2024, most of the patients in the 'self-harm’ and 'ward referrals’ groups identified as White (90.9% and 89.5%, respectively), which is consistent with 2024 census data (91.4%). ‘Mixed or multiple ethnic groups’ were absent in the self-harm group and underrepresented in ward referrals (0.7% vs. 1.3%). ‘Asian, Asian Scottish or Asian British’ individuals (1.7% and 1.5%) and African individuals (0.4% and 0.8%) were also underrepresented compared with census data. The proportion of ‘Caribbean or Black’ individuals is consistent across all groups, aligning with their low representation in the overall population (0.2%). Patients in the ‘Other ethnic groups’ category were slightly overrepresented, highlighting areas for further investigation and intervention.

**Conclusion:** This audit has highlighted significant findings regarding the ethnic representation of patients referred to the Liaison Psychiatry Department at Aberdeen Royal Infirmary. ‘White’ individuals dominate referrals, while ‘Mixed or multiple ethnic groups’, ‘African’, and ‘Asian, Asian Scottish or Asian British’ individuals are notably underrepresented. Conversely, individuals from ‘Other ethnic groups’ are slightly overrepresented. To address these disproportionalities, recommendations include improving ethnicity data collection, comparing the urgency of referrals, fostering community outreach to underrepresented groups, and providing cultural competency training for staff. Further research into systemic and social factors is essential, alongside ongoing monitoring and evaluation of progress. These measures aim to promote equitable, culturally informed mental health services, ensuring inclusive care for all ethnic backgrounds.

